# A potential role for restricted intertactical heritability in preventing intralocus conflict

**DOI:** 10.1111/eva.13292

**Published:** 2021-09-28

**Authors:** Madilyn M. Gamble, Ryan G. Calsbeek

**Affiliations:** ^1^ Graduate Program in Ecology, Evolution, Ecosystems, and Society Dartmouth College Hanover NH USA

**Keywords:** alternative reproductive tactics, antagonistic selection, heritability, intralocus conflict, *Oncorhynchus kisutch*

## Abstract

Intralocus sexual conflict, which arises when the same trait has different fitness optima in males and females, reduces population growth rates. Recently, evolutionary biologists have recognized that intralocus conflict can occur between morphs or reproductive tactics within a sex and that intralocus tactical conflict might constrain tactical dimorphism and population growth rates just as intralocus sexual conflict constrains sexual dimorphism and population growth rates. However, research has only recently focused on sexual and tactical intralocus conflict simultaneously, and there is no formal theory connecting the two. We present a graphical model of how tactical and sexual conflict over the same trait could constrain both sexual and tactical dimorphisms. We then use Coho salmon (*Oncorhynchus kisutch*), an important species currently protected under the Endangered Species Act, to investigate the possibility of simultaneous sexual and tactical conflict. Larger Coho males gain access to females through fighting while smaller males are favored through sneaking tactics, and female reproductive success is positively correlated with length. We tested for antagonistic selection on length at maturity among sexes and tactics and then used parent–offspring regression to calculate sex‐ and tactic‐specific heritabilities to determine whether and where intralocus conflict exists. Selection on length varied in intensity and form among tactics and years. Length was heritable between dams and daughters (*h* ^2^ ± 95% CI = 0.361 ± 0.252) and between fighter males and their fighter sons (0.867 ± 0.312), but no other heritabilities differed significantly from zero. The lack of intertactical heritabilities in this system, combined with similar selection on length among tactics, suggests the absence of intralocus conflict between sexes and among tactics, allowing for the evolution of sexual and tactical dimorphisms. Our results suggest that Coho salmon populations are unlikely to be constrained by intralocus conflict or artificial selection on male tactic.

## 1 INTRODUCTION

The maintenance of diversity between and within the sexes has long captivated biologists. Males and females of most sexually reproducing taxa share most of the same genes yet often exhibit dramatic differences in how those genes are expressed as phenotypes. Differences between the sexes may result from sexually antagonistic selection when the traits conferring high fitness differ between males and females (Bonduriansky & Chenoweth, 2009; Cox & Calsbeek, 2009; Rice & Chippindale, 2001). The ecological and evolutionary consequences of antagonistic selection between the sexes are diverse and can influence standing levels of genetic variation, population growth rate, and intersexual correlations for fitness and other life‐history traits, depending on the strength and direction of intersexual genetic correlations of the traits under antagonistic selection (Bielak et al., 2014; Bonduriansky & Chenoweth, 2009; Foerster et al., 2007; Lande, 1980; Pischedda & Chippindale, 2006; Prasad et al., 2007). Importantly, the degree to which males and females can independently respond to antagonistic selection will depend on the strength and direction of these intersexual genetic correlations (Bonduriansky & Chenoweth, 2009). The evolution of sexual dimorphism may, in some cases, reflect partial resolution of conflict arising from antagonistic selection, and this pattern has largely dominated the study of genomic conflict (Cox & Calsbeek, 2009). However, because sexually dimorphic traits can be genetically correlated with other traits, sex‐dependent trait expression does not always indicate resolved conflict (Cox & Calsbeek, 2009; Harano et al., 2010).

**FIGURE 1:**
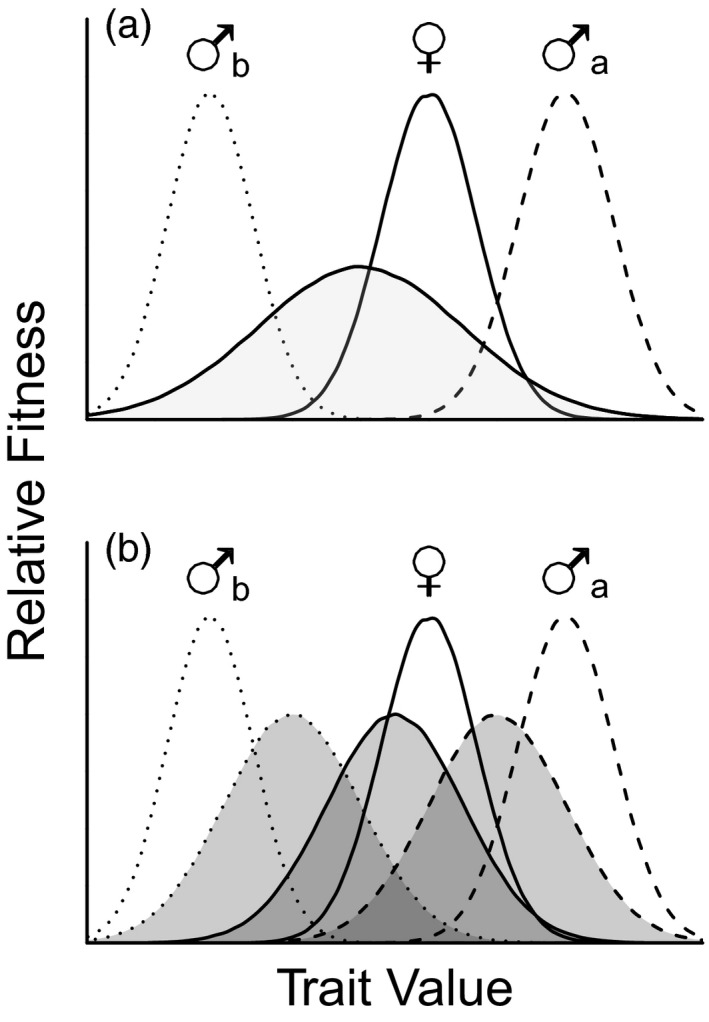
(a) Intralocus tactical and sexual conflicts occur when fitness optima for a trait (unshaded distributions), but not trait expression (shaded distribution), differ between males and females and between male tactics. (b) Sexual and tactical dimorphism can occur without the full resolution of intralocus conflict, which would occur if the trait expression distributions (shaded) fell exactly under the fitness optima distributions (unshaded). Adapted from Cox and Calsbeek (2009)

Conflict arising from antagonistic selection need not be limited to differences in males and females. For example, alternative reproductive tactics (ARTs) face many of the same adaptive challenges as do males and females (Morris et al., 2013): Different phenotypes may emerge from different genotypes (Shuster & Wade, 1991; Sinervo & Lively, 1996; Zimmerer & Kallman, 1989; Zuk et al., 2006) or from identical genotypes mediated by development and the environment (Emlen, 1994; Moczek & Emlen, 1999; Oliveira et al., 2008; Rowland & Emlen, 2009) and may be subject to opposing selection pressures depending on the tactic in which they are expressed (Engqvist & Taborsky, 2016; Hunt & Simmons, 1997; Taborsky & Brockmann, 2010). Increasing appreciation for the parallels between sexual and tactical dimorphisms has led to a handful of recent studies focused on intralocus tactical conflict, which occurs when selection and genetic correlations act in opposite directions between two or more alternative reproductive tactics (Bielak et al., 2014; Buzatto et al., 2015, 2018; Morris et al., 2013). As in the case of sexual dimorphism, the presence of ARTs implies alternative fitness optima within a sex. If this is true, a prediction would be that selection gradients on a common trait should differ among tactics (Figure 1a). As is also the case for sexual dimorphism, the pattern of polymorphism alone is insufficient evidence for the resolution of intralocus conflict among tactics (Cox & Calsbeek, 2009; Figure 1b). For example, recent work has shown that genetic correlations for the same trait between different tactics can limit the independent evolution of each tactic (Abbott & Svensson, 2010; Buzatto et al., 2015, 2018) and that the genes for some alternative male tactics can differentially affect the fitness of their daughters in species as divergent as bulb mites (Bielak et al., 2014) and lizards (Sinervo & Zamudio, 2001). These studies suggest that ARTs may contribute to patterns of genomic conflict.

While intralocus sexual and tactical conflicts have been studied separately, and parallels between sexual and tactical polymorphisms have been recognized, few studies have focused on how these analogous processes might interact in the same system. Specifically, we still do not understand the evolutionary consequences of having multiple fitness peaks both between sexes and among tactics. This is an important shortcoming since antagonistic selection between sexes and among tactics does not occur in isolation—if the genetic architecture for a given trait is shared between the sexes as well as among tactics, then that trait may experience different forms of selection in each context. Thus, for example, the response to selection acting on a trait in females could be influenced by intralocus conflict among male tactics, a pattern that could be easily overlooked if a trait is studied only at the level of sex or male tactic but not both. From this perspective, it may be more useful to consider females, fighter males, and sneaker males as three different tactics rather than treating their differences as two separate variables. Depending on the strength and direction of intersexual and intertactical genetic correlations, antagonistic selection between sexes and tactics could theoretically (1) maintain variation for ARTs if traits that make a low fitness male tactic also make a high fitness female, (2) reduce population growth rate if traits that make a high fitness male tactic also make a low fitness female, and (3) result in reduced or negative genetic correlations for certain traits between sexes and tactics, thus avoiding conflict altogether. Here, we take new steps toward addressing these gaps in understanding, using Salmonid fishes as a study system.

Salmonid fishes are an excellent system in which to investigate the combined effect of sexual and tactical dimorphism. Salmon ARTs are defined by differences in age and length at maturity generally exhibited by males but not females (Quinn, 2018). “Jacks” are faster growing males that mature 1–2 years earlier and considerably smaller than females and other males in the population and employ a sneaking or satellite strategy to gain parentage. “Hooknose” males mature at the same age as (but sometimes larger than) the females and fight to win proximity to females on the spawning grounds. Because most salmonids are semelparous, the tactics represent two solutions to a trade‐off between individual reproductive success and survival to reproduction: Hooknose males spend more time in the ocean compared with jacks, decreasing their chances of surviving to reproduce but increasing their chances of fertilizing eggs if they do survive (Quinn, 2018).

Studies of selection on length at maturity in salmonids have reported inconsistent patterns. In mating experiments, male length at maturity has been shown to be under disruptive selection (Gross, 1985) and negative frequency‐dependent selection (Berejikian et al., 2010; Thomaz et al., 1997). Multiyear studies have revealed temporal variation in the strength and mode of selection. In a 19‐year study, Seamons et al. (2007) found that both male and female Steelhead (*Oncorhynchus mykiss*) experienced stabilizing, disruptive, and positive directional selection on length at maturity in different years, but that larger individuals of both sexes had higher fitness in most years. In a 2‐year study of Coho salmon, Kodama et al. (2012) found positive directional selection on length at maturity for hooknose and jack males in both years, though selection on length was not statistically significant except for in hooknose males in one of the years. Pink salmon (*Oncorhynchus gorbuscha*) also experienced positive selection on length at maturity in males and females across four breeding seasons (Dickerson et al., 2002), though pink salmon do not exhibit male ARTs (Quinn, 2018). Female length at maturity has most often been found to be under directional or stabilizing selection (larger females produce more and larger eggs, with an upper size limit above which they may be unable to reach their spawning grounds; Quinn, 2018). Male age and length at maturity are thought to be influenced by a genetically determined, heritable maturation threshold and environmental conditions that determine whether that threshold is reached (Heath et al., 1994; Lepais et al., 2017). Female Pacific salmon vary less than males do in length and age at maturity and by definition do not mature as young as jack males; nor do females develop the exaggerated jaws and humped backs associated with hooknose males (Quinn, 2018). Therefore, we have no prior expectation about whether sexual conflict arising from ARTs would manifest between females and jacks or hooknose males.

Although males are more variable than females in their life history and morphology, female age, length, fecundity, and egg size still vary considerably within and among populations. Because length at maturity is heritable (median reported narrow‐sense heritability for Salmonine species is 0.21 for both length and age; Carlson & Seamons, 2008) and because longer females have higher fecundity and larger eggs (Quinn, 2018), it is possible that genes carried by jacks could produce daughters that are younger or smaller at maturity—and thus less fit—than those of hooknose males. Alternatively, jacks might pass genes for faster growth to their daughters, and because females do not mature precociously, jacks would have larger and fitter daughters than hooknose males. If there is no intersexual genetic correlation for length at maturity, the genes carried by jacks would not affect their daughters’ length or fitness at all. Unfortunately, most studies on the effects of male salmon ARTs on their offspring do not differentiate between male and female offspring (but see Iwamoto et al., 1984 and Duston et al., 2005), nor do they follow offspring to maturity, precluding conclusions about intersexual correlations for fitness. Even fewer studies have linked male ART to the reproductive success of their daughters.

Understanding how the genetic architecture underlying male ARTs affects females carrying those genes has important implications for conservation, as female fecundity is a limiting factor in population growth (Manning, 1984; Stearns, 1992; Whitlock & Agrawal, 2009). This is especially true in semelparous species such as salmonids (Stearns, 1976). If, for example, jacks have larger and more fecund daughters than hooknose males, selection against jacks in hatcheries would reduce population growth rates. In species that are threatened or endangered, such as Coho salmon, reduced population growth rates would be detrimental to conservation efforts. While recent work has begun to address sexual conflict in salmonids by uncovering the genetic architecture of age and length at maturity and ARTs in salmonids (Ayllon et al., 2015; Barson et al., 2015; Pearse et al., 2019; Sinclair‐Waters et al., 2020), it is clear that species and populations vary in both genetic architecture for these traits and the degree to which that genetic architecture is sex‐specific (Kusche et al., 2017; Waters et al., 2021).

While age and length at maturity are correlated in salmonids, size also varies considerably within age groups, and this variation may still be subject to sex‐ and tactic‐specific selection. Here, we quantify the degree of intralocus sexual and tactical conflict over length at maturity between sexes and tactics in Coho salmon (*Oncorhynchus kisutch*). Given that intralocus tactical conflict and intralocus sexual conflict are essentially identical in concept and computation (Abbott et al., 2019), we henceforth use the term “tactic” to refer to females, jacks, and hooknose males. Using a 2‐generation pedigree of wild Coho, we first determined whether selection was tactically antagonistic by quantifying tactic‐specific selection gradients for length at maturity. We then estimated intertactical heritability through tactic‐specific single parent–offspring regressions to determine whether length at maturity could evolve independently in each tactic. We compared intertactical heritabilities with selection gradients to determine whether intralocus conflict exists among females, hooknose males, and jacks. Finally, we use our results to address the possible effects of intralocus sexual and tactical conflict on the maintenance of genetic variance for ARTs, population growth rate, and inter‐ and intrasexual correlations for fitness. Given existing sexual and tactical dimorphism in this species (Quinn, 2018), we hypothesized that selection gradients and parent–offspring correlations would differ among females, jacks, and hooknose males.

## 2 METHODS

### 2.1 Data

We used a publicly available dataset containing pedigreed sex‐specific life history and lifetime reproductive success data (Banks et al., 2013) for a population of Coho salmon (*O*. *kisutch*) from Calapooya Creek, Oregon, USA. The dataset is described in detail by Thériault et al. (2011) and was originally collected to compare the reproductive success of fish bred and reared in hatcheries versus in the wild. Briefly, sexually mature fish were sampled as they returned to the river to spawn between 2001 and 2009 and a 2‐generation pedigree was constructed using 10 microsatellite loci and a maximum‐likelihood‐based parentage analysis as described in Thériault et al. (2011). The pedigree included data on reproductive success and length for both a parent generation and an offspring generation, allowing for selection analysis on length at maturity for two generations and for the calculation of heritability of length at maturity. Reproductive success of each individual was measured as the number of their offspring that returned as adults to spawn. As such, this metric includes components of both offspring survival and parent reproduction, which unfortunately could not be avoided with this dataset. Thériault et al. (2011) classified male salmon as 2‐year‐old “jacks” if they were below 500 mm length and as 3‐year‐old hooknose males based on a bimodal length distribution for males; we retain their classification of male tactic in our analyses. We used only fish that (1) were spawned and reared in the wild, (2) were assigned to both a dam and a sire that were also reared in the wild, and (3) had both length and reproductive success data. The final dataset included a total of 1398 individual wild Coho salmon (681 females and 717 males). Of these, 775 were offspring (380 females and 395 males) captured between 2004 and 2006 with known sires and dams. The 623 parents of these fish included 301 dams and 322 sires (264 hooknose and 58 jack males) caught in 2002 and 2003. Hooknose males sired 678 of the offspring (345 sons and 333 daughters) and jack males sired 97 of the offspring (50 sons and 47 daughters). Although age at maturity is correlated with length in salmonids (Quinn, 2018), and thus, length at age would also be of interest, tactic and age are completely conflated in this dataset: All jacks were two years old, while all females and hooknose males were three years old. While there was variation in length at maturity within and between tactics, the only variation in age at maturity was between tactics. Therefore, we did not include age in our analyses.

### 2.2 Selection analysis

We used ANOVA to compare length at maturity among females, hooknose males, and jacks. Because the dataset included 5 years of reproductive success data (2002–2006), we first determined whether selection on length at maturity, and any differences in selection among tactics, differed among years. To do this, we fit a generalized linear model (*glmz*) with a Poisson family and a log‐link relating absolute reproductive success to standardized length, the square of standardized length, tactic, year, and all interactions involving length:

$$\begin{array}{cc} \text{RS} & =\beta_{0}+\beta_{1}\left(\text{StdLength}\right)+\beta_{2}{\left(\text{StdLength}\right)}^{2}+\beta_{3}\left(\text{Tactic}\right)+\beta_{4}\left(\text{Year}\right)+\beta_{5}\left(\text{StdLength*Tactic}\right)\\ & \;+\beta_{6}\left({\text{StdLength}}^{2}*\text{Tactic}\right)+\beta_{7}\left(\text{StdLength*Year}\right)+\beta_{8}\left({\text{StdLength}}^{2}*\text{Year}\right)+\beta_{9}\left(\text{Tactic*Year}\right)\\ & \;+\beta_{10}\left(\text{StdLength*Tactic*Year}\right)+\beta_{11}\left({\text{StdLength}}^{2}*\text{Tactic*Year}\right)+\varepsilon \end{array}$$

Significant 2‐way interactions between year and standardized length or the square of standardized length would suggest that linear or quadratic selection differed among years. Significant 3‐way interactions with year would indicate that linear or quadratic selection differed among tactics in ways that differed among years. We then analyzed selection on length at maturity, first for all years pooled and then separately for each year. For each year and for the pooled data, we also analyzed selection for all tactics pooled and separately for each tactic. To determine the statistical significance of linear and quadratic selection on length in each situation, we fit a *glmz* with a Poisson family and a log‐link relating absolute reproductive success to standardized length and the square of standardized length. Length was standardized to the population mean in units of standard deviations (Arnold & Wade, 1984). Significance of each term was determined using analysis of deviance with a chi‐square test.

We quantified natural selection on length using traditional selection gradient analyses with length and the square of length standardized to the population mean in units of standard deviations (Arnold & Wade, 1984). The population mean was calculated for each selection analysis based on the portion of the population being considered: For selection analysis on all years and tactics pooled, we used the mean of all years and tactics; for selection analyses on a given year with all tactics pooled, we used the mean of all individuals that reproduced in that year, regardless of tactic; and for selection analyses on a given tactic within a year, we used the mean of all individuals of that tactic reproducing in that year. We measured relative reproductive success by dividing each individual's reproductive success by the population mean (Lande & Arnold, 1983). We estimated linear (i.e., directional) selection as the regression coefficient (β ± 1SE) for relative reproductive success as a function of standardized length:

$$w=\beta_{0}+\beta_{1}*\text{Stdlength}+\varepsilon$$

We estimated quadratic (i.e., stabilizing or disruptive) selection from the partial regression coefficient for relative reproductive success as a function of the square of standardized length. These quadratic models also included linear terms:

$$w=\beta_{0}+\beta_{1}*\text{StdLength}+\gamma *{\text{StdLength}}^{2}+\varepsilon$$

Estimates of quadratic selection (γ ± 1SE) are calculated by doubling the quadratic regression coefficient and its associated standard error (Phillips & Arnold, 1989). Although selection gradients can be calculated directly from the coefficients of the *glmz* (Chevin et al., 2015; Morrissey & Goudie, 2016), these methods perform about as well as standard OLS regression methods originally proposed by Lande and Arnold (1983) and Arnold and Wade (1984) (Morrissey & Goudie, 2016). For simplicity, we used OLS regression for estimates of selection (Lande & Arnold, 1983). We visualized fitness functions using cubic splines implemented through the “gam” function in R (Schluter, 1988). We performed all statistical analyses in R.

### 2.3 Parent–offspring regression

Recent advances in the field of quantitative genetics, particularly the use of animal models (Kruuk, 2004; Wilson et al., 2010), have allowed for more robust estimates of quantitative genetic parameters such as additive genetic variance and covariance. The advantage of these models over parent–offspring regression is that they make use of pedigree relationships other than those between parents and offspring (Kruuk, 2004). However, the benefits of animal models can only be realized with much larger sample sizes (often thousands of individuals) and denser pedigrees (i.e., individuals related in ways other than parent–offspring) compared with those required by traditional parent–offspring regression (Kruuk, 2004). Because of the comparatively low sample size of this pedigree, the low number of maternal half‐siblings per individual (mean ± SD = 1.8 ± 3.9), and because preliminary animal model analyses to estimate additive genetic (co)variances for length at maturity exhibited very poor mixing and failed to converge, we used traditional parent–offspring regression rather than animal models and estimated intertactical heritabilities instead of intertactical genetic correlations. In the absence of the ability to measure genetic correlations, intertactical heritability estimates of length at maturity still indicate whether selection on one tactic could drive evolution of another. Specifically, an intertactical heritability of 1 would represent complete evolutionary constraint (at least until the G matrix evolves), while a genetic correlation greater than 0 represents partial constraint (slower evolution toward the optimal phenotype than would be achieved with a genetic correlation of 0). Thus, we tested the hypotheses that (1) single parent–offspring regression slopes differ from zero (i.e., intertactical heritabilities differ from 0) and (2) single parent–offspring regression slopes differ from 0.5 (i.e., heritabilities differ from 1). In instances where parent and offspring tactic variances were unequal, we tested the null hypothesis that the single parent–offspring regression slopes differ from 0.5/(parent standard deviation/offspring standard deviation; i.e., heritabilities differ from 1; Falconer & Mackay, 1989, p. 168).

To determine the intertactical heritabilities of length at maturity, we used a linear model relating mean offspring length to dam length, sire length, offspring tactic, and two‐way interactions between dam length and offspring tactic and sire length and offspring tactic:

$$\begin{array}{cc} \text{OffspringLength} & =\beta_{0}+\beta_{1}\left(\text{DamLength}\right)+\beta_{2}\left(\text{SireLength}\right)+\beta_{3}\left(\text{OffspringTactic}\right)\\ & \;+\beta_{4}\left(\text{DamLength*OffspringTactic}\right)+\beta_{5}\left(\text{SireLength*OffspringTactic}\right)+\varepsilon \end{array}$$

A significant interaction would show that the correlation between parent and offspring length differed depending on whether the offspring was a female, jack, or hooknose male; such a result would suggest that the heritability of length at maturity differs among tactics and would need to be calculated independently. Nonsignificant interactions would indicate that the slope of the parent–offspring regression for length does not differ among tactics and that a single heritability estimate would apply to all tactics. Because single parent–offspring regressions only give a valid estimate of heritability if the phenotypic variances of the trait in question are equal between the sexes (Falconer & Mackay, 1989)—or in this case, tactics—we used a Bartlett test to determine whether the variance in length at maturity differed among tactics before calculating tactic‐specific heritabilities. We did not include maternal or environmental affects in these models, both because of limited power due to low sample size and because maternal effects on length in salmonids are thought to disappear early in life (Silverstein & Hershberger, 1992, 1994). Heritabilities were estimated by multiplying the slope of the single parent–offspring regression (*b_op_*) by two (Falconer & Mackay, 1989), except in cases where the variances of the parent and offspring tactics were unequal. In this case, heritability was estimated by multiplying the slope of the single parent–offspring regression (*b_op_*) by two times the ratio of the standard deviations of length at maturity for the parent tactic to that of the offspring tactic (2*σ_p_*/*σ_o_*; Falconer & Mackay, 1989, p. 168). Statistical analyses were performed in R version 4.0.3 or JMP Pro 15.

## 3 RESULTS

### 3.1 Selection analysis

The length of females (dams and daughters) ranged from 520 to 875 mm (mean ± SE: 733.06 ± 1.84 mm). Males (sires and sons) ranged in length from 140 to 890 mm (mean ± SE: 677.95 ± 5.187 mm. Jacks ranged in length from 140 to 495 mm (mean ± SE: 423.45 ± 3.39 mm), and hooknose males ranged from 530 to 890 mm (mean ± SE: 735.38 ± 6.29 mm). Length differed significantly among tactics (*F*_2,1395_ = 1671, *p* < 0.0001). A Tukey's HSD test showed that jacks were significantly shorter in length than females (*p* < 0.0001) but length did not differ between females and hooknose males (*p* = 0.8).

**FIGURE 2:**
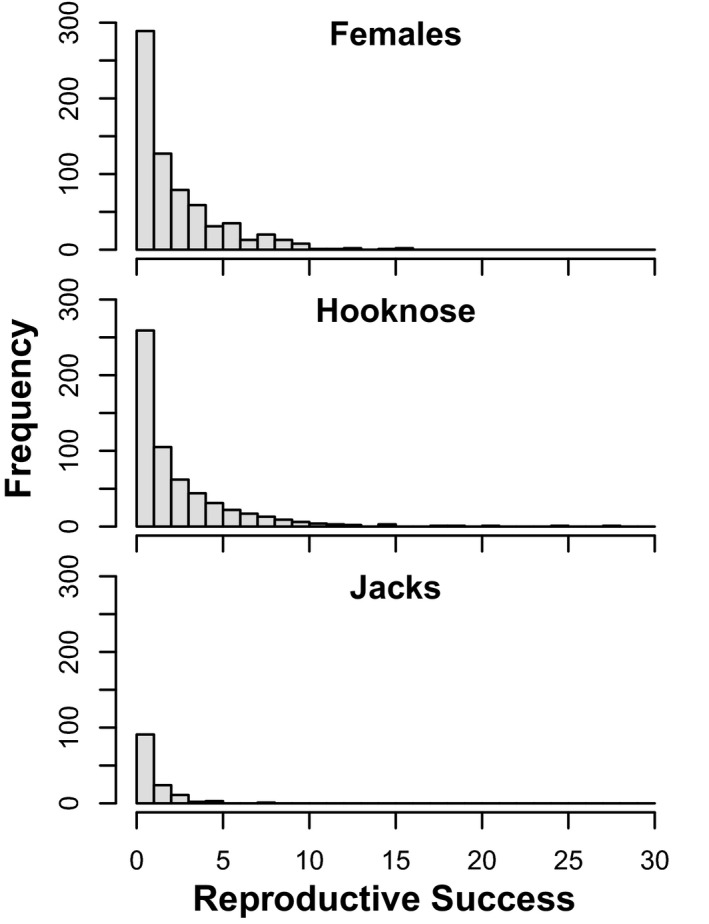
The distribution of individual reproductive success (number of offspring surviving to sexual maturity) for each tactic (female, hooknose male, and jack male)

Female reproductive success ranged from 0 to 16 offspring surviving to maturity (mean ± SE: 2.61 ± 0.10; Figure 2). Male reproductive success ranged from 0 to 28 (mean ± SE: 2.51 ± 0.11) offspring surviving to maturity. Within males, the reproductive success of hooknoses ranged from 0 to 28 (mean ± SE: 2.80 ± 0.14) while the reproductive success of jack males ranged from 0 to 8 (mean ± SE: 1.21 ± 0.11). A negative binomial generalized linear model revealed that reproductive success differed significantly among tactics, χ^2^(*df* = 2, *N* = 1398) = 54.19, *p* < 0.0001.

There was no significant 3‐way interaction between standardized length squared, tactic, and year, χ^2^(*df *= 2, *N* = 1398) = 2.98, *p* = 0.2, but the 3‐way interaction between standardized length, tactic, and year was marginally significant, χ^2^(*df *= 2, *N* = 1398) = 5.34, *p* = 0.07. Removing the former 3‐way interaction rendered the latter insignificant, χ^2^(*df *= 2, *N* = 1398) = 2.47, *p* = 0.3, we removed this to determine the significance of the 2‐way interactions. There was a significant interaction between standardized length and year, χ^2^(*df *= 1, *N* = 1398) = 33.66, *p* < 0.0001 and between standardized length squared and tactic, χ^2^(*df *= 2, *N* = 1398) = 6.87, *p* = 0.03, suggesting that linear selection differed among years and quadratic selection differed among tactics.

**FIGURE 3:**
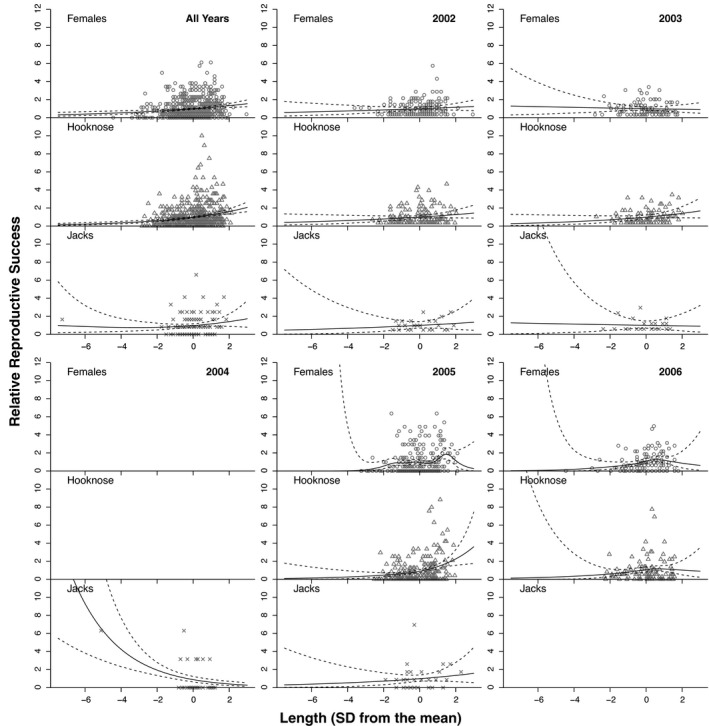
Scatterplots and cubic splines relating relative reproductive success to standardized length for each tactic (females, jacks, and hooknose males) and each year. Blank plots indicate no individuals of that tactic returned in that year. Dashed lines show 2SE

When all years were pooled, linear selection was strongest in hooknose males (β = 0.239 ± 0.047), intermediate in females (β = 0.142 ± 0.039), and weakest in jacks (β = 0.098 ± 0.093; Table 1). Quadratic selection was positive but weak in hooknose males (γ = 0.007 ± 0.081) and in jack males (γ = 0.086 ± 0.050) and was negative and weak in females (γ = −0.038 ± 0.050). In the year‐by‐year analyses, selection generally favored longer or intermediately sized females (positive linear selection and negative quadratic selection; Table 1, Figure 3). The same was true for hooknose males, though the linear component of selection was stronger than that in females in three out of four years. Linear and quadratic selection on length at maturity in jacks were each positive in two years and negative in two years; none of these selection gradients were significantly different from zero (Table 1, Figure 3).

**TABLE 1:** Linear (β) and quadratic (γ) selection gradients on length and their statistical significance for each year and tactic (females, hooknose, and jack males)

Year	Tactic	β ± SE	χ^2^ (*df*, *N*)	*p*	γ ± SE	χ^2^ (df, *N*)	*p*
All	All	**0.249 ± 0.029**	171.48 (1, 1398)	<0.0001	0.111 ± 0.044	2.34 (1, 1398)	0.1
	Female	**0.142 ± 0.039**	41.27 (1, 681)	<0.0001	**−0.038 ± 0.050**	4.46 (1, 681)	0.03
	Hooknose	**0.239 ± 0.047**	83.70 (1, 585)	<0.0001	0.007 ± 0.081	1.72 (1, 585)	0.2
	Jack	**0.098 ± 0.093**	4.56 (1, 133)	0.03	**0.086 ± 0.050**	3.60 (1, 133)	0.06
2002^a^	All	**0.115 ± 0.040**	9.31 (1, 391)	0.002	0.043 ± 0.059	0.42 (1, 391)	0.5
	Female	0.071 ± 0.054	2.30 (1, 192)	0.1	0.015 ± 0.068	0.033 (1, 192)	0.9
	Hooknose	**0.119 ± 0.066**	7.96 (1, 167)	0.005	−0.079 ± 0.116	1.39 (1, 167)	0.2
	Jack	0.100 ± 0.102	0.01 (1, 32)	0.9	−0.027 ± 0.227	0.02 (1, 32)	0.9
2003^a^	All	**0.158 ± 0.048**	8.42 (1, 232)	0.004	0.025 ± 0.082	0.00 (1, 232)	>0.9
	Female	−0.034 ± 0.071	0.16 (1, 109)	0.7	−0.150 ± 0.115	2.90 (1, 109)	0.09
	Hooknose	0.175 ± 0.074	0.92 (1, 97)	0.3	0.192 ± 0.121	2.77 (1, 97)	0.1
	Jack	−0.033 ± 0.126	0.20 (1, 26)	0.7	−0.120 ± 0.233	0.19 (1, 26)	0.7
2004^b^	All	NA	NA	NA	NA	NA	NA
	Female	NA	NA	NA	NA	NA	NA
	Hooknose	NA	NA	NA	NA	NA	NA
	Jack	−0.837 ± 0.253	1.06 (1, 41)	0.3	0.222 ± 0.201	0.070 (1, 41)	0.8
2005^c^	All	**0.291 ± 0.062**	117.97 (1, 503)	<0.0001	0.262 ± 0.086	18.04 (1, 503)	<0.0001
	Female	**0.221 ± 0.085**	24.56 (1, 254)	<0.0001	0.012 ± 0.117	0.50 (1, 254)	0.5
	Hooknose	**0.378 ± 0.094**	67.25 (1, 216)	<0.0001	0.297 ± 0.173	3.52 (1, 216)	0.06
	Jack	0.160 ± 0.242	0.14 (1, 33)	0.7	0.122 ± 0.440	0.10 (1, 33)	0.7
2006^d^	All	**0.190 ± 0.076**	22.78 (1, 231)	<0.0001	**−0.111 ± 0.102**	12.60 (1, 231)	<0.001
	Female	**0.218 ± 0.089**	8.50 (1, 126)	0.004	**−0.062 ± 0.097**	9.06 (1, 126)	0.003
	Hooknose	**0.164 ± 0.127**	8.75 (1, 105)	0.003	**−0.203 ± 0.227**	7.10 (1, 105)	0.008
	Jack	NA	NA	NA	NA	NA	NA

Note

Selection gradients are presented with their standard errors; bold font indicates statistical difference from zero. Chi‐square test statistics (χ^2^) are presented with degrees of freedom (*df*) and sample size (*N*).

^a^ Parent generation.

^b^ Only jacks (brood year (BY) 2002).

^c^ Jacks from BY 2003; females and hooknose males from BY 2002.

^d^ Only females and hooknose males (BY 2003).

### 3.2 Parent–offspring correlations

We found a significant 2‐way interaction between sire length and offspring tactic (*F*_2,765_ = 4.15, *p* = 0.016). We proceeded by running linear models relating offspring length to parent length separately for each parent and offspring tactic to estimate single parent–offspring heritabilities between and within each tactic (Table 2). Variance in length differed among tactics (Bartlett's *K*‐*squared* = 77.85, *df* = 2, *p* < 0.0001), so we used *F* tests to compare pairwise variances between tactics. Variance in length at maturity varied significantly between hooknose males and females (*F*_379,320_ = 0.415, *p* < 0.0001) and between hooknose males and jacks (*F*_320,73_ = 2.77, *p* < 0.0001), but not between females and jacks (*F*_379,73_ = 1.15, *p* = 0.47). Thus, we estimated intertactical heritabilities between hooknose males and females and between hooknose and jack males by multiplying the slope of the single parent–offspring regression (*b_op_*) by two times the ratio of the standard deviations of length at maturity for the parent tactic to that of the offspring tactic (2*σ_p_*/*σ_o_*; Falconer & Mackay, 1989, p. 168; see Table 2 for *h^2^* calculations). The intratactical heritabilities, and the intertactical heritabilities between females and jacks, were estimated by doubling *b_op_* (Falconer & Mackay, 1989).

**TABLE 2:** Coefficients and statistical parameters of single parent–offspring regressions for length at maturity

Model (Offspring~Parent)	Slope estimate ± SE	H_0_: slope = 0		H_0_: slope = 0.5		*h* ^2^ ± 95%CI^a^
		*t*	*p*	*F*_(_ *_df_*_)_	*p*	
Daughter~Dam	0.181 ± 0.064	2.812	0.005	24.6_(1,222)_	<0.0001	0.361 ± 0.252
Jack~Dam	−0.052 ± 0.010	−0.538	0.593	32.1_(1,59)_	<0.0001	−0.105 ± 0.383
Hooknose~Dam	−0.070 ± 0.106	−0.656	0.512	63.4_(1,203)_	<0.0001	−0.090 ± 0.268
Daughter~Jack	0.083 ± 0.249	0.334	0.741	2.9_(1,16)_	0.1	0.166 ± 0.975
Jack~Jack	0.233 ± 0.249	0.934	0.366	1.2_(1,14)_	0.3	0.465 ± 0.976
Hooknose~Jack	−0.051 ± 0.466	−0.109	0.914	3.6_(1,27)_	0.005	−0.061 ± 1.10
Daughter~Hooknose	0.060 ± 0.050	1.207	0.229	27.6_(1,172)_	<0.0001	0.187 ± 0.303
Jack~Hooknose	−0.045 ± 0.084	−0.537	0.594	17.0_(1,44)_	0.0002	−0.150 ± 0.546
Hooknose~Hooknose	0.433 ± 0.080	5.444	<0.0001	0.7_(1,169)_	0.4	0.867 ± 0.312

Note

*p* Values associated with *t* statistics and *F* statistics refer to tests of the hypothesis that regression slopes are different from 0 and 0.5, respectively (i.e., heritability of 0 and 1), except in cases where the variance in length at maturity differs between parent and offspring tactics. In these cases, the null hypothesis is that the slope of the single parent–offspring regression = 0.5/(parent standard deviation/offspring standard deviation).

^a^ Heritability estimates (*h* ^2^) are calculated either as 2*b_op_* or as 2*b_op_* *(*σ_p_*/*σ_o_*).

**FIGURE 4:**
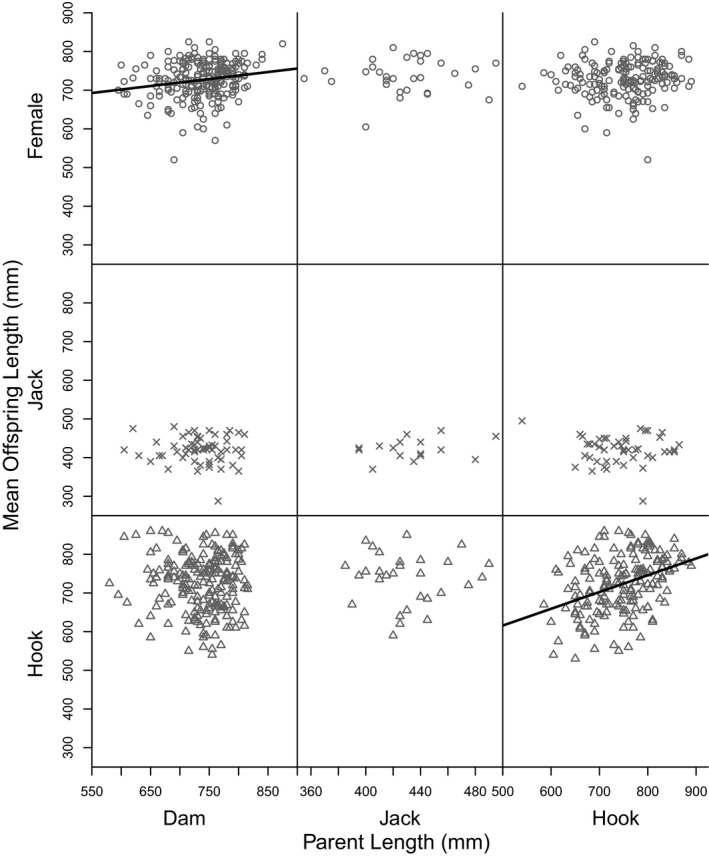
Single parent–offspring regressions for length between dams, jack sires, and hooknose sires and their offspring. Offspring tactic is represented by an (x) for jacks, an (o) for females, and a triangle for hooknose males. Significant parent–offspring relationships are represented by black regression lines; no regression lines are drawn for insignificant relationships. Note different scales of *x*‐, but not *y*‐axes

The only parent–offspring heritabilities that were statistically different from zero were those between dams and daughters and hooknose sires and hooknose sons (Table 2; Figure 4). The hooknose sire‐hooknose son heritability estimate (*h* ^2^ ± 95%CI = 0.867 ± 0.312) was almost 2.5 x greater than the dam‐daughter heritability estimate (0.361 ± 0.252). The jack sire‐jack son estimate (0.465 ± 0.976) was intermediate between dam‐daughter and hooknose sire–hooknose son estimates, but was not significant. All parent–offspring heritabilities were significantly different from one except for those between daughters and jacks, jack sons and jack sires, hooknose sons and jack sires, and hooknose sons and hooknose sires (Table 2).

## 4 DISCUSSION

### 4.1 Tactic‐specific selection and intralocus conflict

Intralocus conflict occurs when the same trait is under different selection pressures in two different genetic backgrounds (i.e., sexes or tactics) and is genetically correlated in those genetic backgrounds (Rice & Chippindale, 2001). While dimorphism can occur with the breakdown of genetic correlation and alleviate intralocus conflict, dimorphism itself is not evidence that conflict has been fully resolved (Cox & Calsbeek, 2009). Our results suggest that any intralocus conflict that may have existed over length at maturity between females, jacks, and hooknose males has been resolved. This is evident in that most intertactical heritabilities are less than one, and all intertactical heritabilities are not significantly different from zero. The lack of intertactical heritability for length at maturity suggests a breakdown of intertactical genetic correlations, allowing each tactic to evolve independently in response to selection on length. This is reflected in the clear tactical dimorphism in Coho salmon observed in nature. The lack of intertactical heritability is consistent with results from other studies examining differences between sexes (Lehtovaara et al., 2013) or ontogenetic life stages (Goedert & Calsbeek, 2019) and suggests a means by which alternative tactics can achieve independent fitness optima.

Linear and quadratic selection varied across years and tactics. Across years, visualizations of selection on length showed that females experience both stabilizing and positive directional selection, hooknose males experience mostly positive directional selection, and jacks experience very weak selection. When we pooled all years, the linear selection coefficient for hooknose males was 68% greater than that for females, which in turn was 45% greater than the linear selection coefficient for jacks. Although we cannot make strong conclusions about selection on jack length given the small sample size within years, our results suggest that selection on length is not antagonistic among tactics. Thus, even if we had found evidence of heritability for length among tactics, the lack of antagonistic selection precludes intralocus conflict over length at maturity. The yearly variation in the strength of linear selection, and the pattern of stronger selection on males than in females, is consistent with studies of other salmonids (Seamons et al., 2007). Studies of other wild animal populations have also found that selection varies by year (pied flycatchers *Ficedula hypoleuca* (Visser et al., 2015), tree crickets *Oecanthus nigricornis* (Ercit, 2016), oystercatchers *Haematopus ostralegus ostralegus* (Pol et al., 2010), soay sheep *Ovis aries* (Milner et al., 1999)).

It is possible that the nonsignificant jack–jack heritability for length and nonsignificant selection gradients on jack length result from small sample sizes of jacks in our dataset. While jacks are commonly undercounted in salmon surveys due to their small size and satellite tactics (Quinn, 2018), the dataset used in this study comes from a research program in which all returning spawners were captured at a dam, meaning all jacks were sampled (Thériault et al., 2011). Thus, our small sample size for jacks is representative of their lower frequency in the population. Our estimate of jack–jack heritability was intermediate between the daughter‐dam and hooknose–hooknose heritability estimates but was highly uncertain. A larger sample size for jacks might have provided evidence that length at maturity is heritable between jacks and their jack sons. It is also possible that a larger sample size may have revealed significant quadratic selection on jack length; alternatively, length could be at its adaptive optimum in jacks.

Although we were not able to account for maternal effects in our heritability estimates, studies of maternal effects on length at maturity in Pacific salmon generally suggest that maternal effects are evident early in the offspring's life but disappear through ontogeny. For example, in one study of Coho salmon, correlation between egg size and offspring size disappeared after 5 months (Silverstein & Hershberger, 1992). In another Coho study, heritabilities for length based only on the dam component of variance decreased consistently between 5 and 11 months posthatching (Silverstein & Hershberger, 1994). In a study of Chinook salmon, maternal effect on body size (weight) disappeared by 200 days after emergence (Heath et al., 1999), and another study found no significant maternal effect found on jacking rate (Forest et al., 2016). In concordance with our study, Iwamoto et al. (1984) found that, in Coho salmon reared for one year, maternal effects explained the variation in length of female offspring, while paternal effects explained more variation in the length of male offspring. We are not aware of any studies on maternal effects that followed both male and female offspring to maturity, making connections to this study difficult.

Even though male tactic is heritable and contributes to variation in male reproductive success in Pacific and Atlantic salmon (Appleby et al., 2003; Berejikian et al., 2010; Duston et al., 2005; Garant et al., 2003; Gjerde & Gjedrem, 1984; Heath et al., 1994, 2002; Iwamoto et al., 1984; Piché et al., 2008; Quinn, 2018), our results suggest that male tactic had no effect on the length of their daughters. This is a surprising result because in addition to male tactic, age at maturity—which is strongly correlated with length at maturity in both sexes—is also heritable in salmonids (Carlson & Seamons, 2008). Moreover, the genes encoding the heritable maturation threshold are located on autosomes (at least in Atlantic salmon; Lepais et al., 2017) and are thus likely to be inherited equally by both sexes. It is reasonable to think that these genes could affect female life history (age and length at maturity) which could in turn affect their reproductive success due to the positive relationship between female length and fecundity and egg size (Quinn, 2018). Alternatively, the genes affecting male maturation threshold could alter fecundity, egg size, or overall maternal investment directly. Although female length is positively correlated with both egg size and number, sire tactic could plausibly explain additional variance in the relationship between maternal investment and female length if the genetic maturation threshold and maternal investment are genetically correlated. Despite these possible avenues for conflict, our results suggest that a male's tactic does not affect his daughters’ length at maturity.

The few studies that have considered heritability of length at maturity and age at maturity separately for male and female salmon illustrate the importance of considering intersexual parent–offspring correlations. Gjerde and Gjedrem (1984) found that the heritability of length calculated from the dam component of variance was higher than that from the sire component in both Atlantic salmon (*Salmo salar*; $h_{\text{dam}}^{2}$ = 0.60 and $h_{\text{sire}}^{2}$ = 0.35) and rainbow trout (*O*. *mykiss*; $h_{\text{dam}}^{2}$ = 0.44 and $h_{\text{sire}}^{2}$ = 0.16). These conclusions suggest that average offspring length and age at maturity were more related to dam traits than sire traits, though neither our study nor theirs was able to account for maternal effects, which could inflate estimates of heritability between dams and their offspring. However, our study includes only fish that matured, while the Gjerde and Gjedrem study sampled all fish at a certain age (2.5–3 years old) regardless of maturity. Gjerde and Gjedrem's study also used only the 220 largest offspring from each full‐sib family, artificially reducing variance in length. Thus, their heritability estimates are not directly comparable to ours, but still demonstrate that heritabilities can differ between the sexes. A study on Chinook salmon (*O*. *tshawytscha*) found that maturation age was less heritable for females than for males ($h_{\text{females}}^{2}$ = 0.39 and $h_{\text{males}}^{2}$ = 0.49; Hankin et al., 1993). This result aligns with our findings on length at maturity, as length and age at maturity are correlated (Quinn, 2018). Our analyses of tactic‐specific parent–offspring heritabilities illustrate the additional insights gained by considering both intersexual and intertactical parent–offspring correlations simultaneously.

Recently, studies have begun to recognize the potential importance of sexual and tactical conflict in salmon, and many have investigated the genetic architecture of age at maturity. These studies have used genome‐wide association studies (GWAS) and single‐nucleotide polymorphism (SNP) analyses to identify genes and genomic regions associated with age at maturity in salmon, which differ across species and populations. In Atlantic salmon, European populations appear to escape sexual conflict in age at maturity through sex‐specific dominance at the *vgll3* and *six6* genes (Ayllon et al., 2015; Barson et al., 2015; Sinclair‐Waters et al., 2020), although this pattern is not found in North American populations of Atlantic salmon (Kusche et al., 2017). The genetic architecture of age at maturity in Pacific salmon is more complex. The *vgll3* gene associated with age at maturity for Atlantic salmon does not appear to influence age at maturity in Chinook, Coho, Sockeye, or Steelhead (Waters et al., 2021). The same study showed that the *six6* gene is associated with age at maturity in Sockeye and Steelhead, although the effect of the *six6* genotype did not differ between sexes in either species (Waters et al., 2021). There was no association between the *six6* gene and age at maturity in Chinook or Coho. A different study of Rainbow Trout (the same species as Steelhead Trout) found some genomic regions associated with early maturation that affected both sexes and others that were sex‐specific (Haidle et al., 2008). Sex‐specific haplotypes associated with age at maturity have been identified for Chinook salmon (McKinney et al., 2021), which would avoid intralocus sexual and tactical conflicts in that species. In Coho salmon, Kodama et al. found genomic regions associated with growth and age at maturity affected males and females differently, though statistical power was low due to few families and small sample sizes within families (Kodama et al., 2018). Collectively, these studies suggest that the genetic architecture for age at maturity in salmonids may differ between the sexes, but that this difference varies among populations and species.

### 4.2 Evolutionary implications

The degree of intralocus conflict in any system has implications for the extent to which the sexes (or tactics) can evolve independently of one another, and thus contributes to the maintenance of variation (Foerster et al., 2007; Hall et al., 2010). The lack of evidence for intralocus sexual and tactical conflicts evinced by our results suggests that, in this system, each sex and tactic may be able to respond separately to selection on length at maturity. Because the daughters of each tactic do not differ with respect to length at maturity, it is likely that neither intralocus sexual nor tactical conflict plays a role in the maintenance of variation between sexes and among tactics in this system. However, the role of intralocus conflict—or the lack thereof—in maintaining alternative reproductive tactics would be better addressed with a pedigree with more than 2 generations, as it is still possible that females carrying genes for each tactic buffer those traits from selection. Additionally, it is possible that differences in fecundity, egg size, or maternal investment between daughters of jacks and hooknose males might contribute to the maintenance of intersexual variation and intrasexual variation. Of course, in species for which ARTs are entirely environmentally determined, intralocus conflict is unlikely to affect their evolution. The lack of intersexual and intertactical heritabilities for length at maturity also means that antagonistic selection does not reduce population growth rates in this system, as it would have if either male tactic produced smaller daughters than the other. Again, though, this cannot be entirely ruled out without data on the relative fecundity, egg size, or maternal investment in daughters of each tactic.

While our results suggest that selection does not differ significantly among tactics, the measure of fitness used in this study is one in which components of offspring fitness (offspring survival to reproductive maturity) are attributed to the parents. Because of this limitation, our measure of fitness assumes offspring survival is causally related to parental phenotype, which might bias our estimates of selection on length at maturity (Grafen, 1988; Wolf & Wade, 2001). As a result, any future analyses that incorporate these data (e.g., meta‐analyses) should take this into account. However, regardless of selection on length at maturity, the lack of intertactical and intersexual heritability of length at maturity suggest that neither intralocus tactical nor sexual conflict is constraining its evolution.

### 4.3 Conservation implications

Salmon hatcheries have often excluded jacks from their broodstock and instead have selected for older, larger males (McLean et al., 2005; Quinn, 2018). Because the growth environment is greatly enhanced in hatcheries compared with wild populations, and because faster freshwater growth leads to earlier maturation in salmon (Larsen et al., 2006; Quinn, 2018; Vøllestad et al., 2004), jacks are still often produced in hatchery populations (Beckman & Larsen, 2005; Larsen et al., 2004, 2013; Unwin & Glova, 1997). However, because maturation threshold is genetically determined and highly heritable (*h* ^2^ = 0.9, (Lepais et al., 2017), and because growth rate is heritable in salmon (Carlson & Seamons, 2008; Kristjánsson et al., 2020; Silverstein & Hershberger, 1995), artificial selection against jacks may still alter the genetic variance for age and length at maturity within hatchery broodstocks. Our results show that selection on male length and tactic in Coho salmon does not have indirect consequences on female length, and thus fecundity and population growth, because sire length and tactic are unrelated to the length of their daughters. If size was heritable between jacks and their daughters, selection against jacks could have resulted in larger females in the population. On the other hand, if jacks had larger daughters than did hooknose males (as they might if growth rate rather than size were heritable between jacks and their daughters), selection against jacks could have decreased female size and thus the number of eggs (Quinn, 2018) produced in hatchery populations. Given the low estimate of heritability in length at maturity between jacks and their daughters, our results suggest that neither of these consequences are likely to have occurred. On the other hand, because life‐history diversity provides population resilience (Hilborn et al., 2003; Healey, 2009; Schindler et al., 2015), some hatchery management plans have recently begun to prioritize the maintenance of life‐history diversity (Anderson et al., 2020), which may include re‐integrating jacks into breeding programs (California HSRG, 2012; CDFG‐NOAA, 2001; Mobrand et al., 2004). Again, given the lack of heritability for length at age between jacks and their daughters, efforts to increase life‐history diversity in threatened populations are unlikely to alter female length at maturity and thus fecundity and population growth rate.

### 4.4 Future directions

Given the threshold nature of age at maturity (Lepais et al., 2017), environmental variation will also play a large role in determining whether males mature as jacks or hooknose males. Future studies should consider the role of environmental factors such as temperature, food availability, and food quality on sex‐ and tactic‐specific parent–offspring relationships. The Pacific Decadal Oscillation (PDO) is an environmental index that indicates whether the marine growth environment (where salmon evaluate whether they have surpassed the maturation threshold) is good or bad for salmon growth. However, between 1998 (when the first parent generation in our dataset was born) and 2007 (when the F2 offspring generation returned to spawn), the PDO was in a “negative” phase, indicating good marine growth conditions for salmon along the Washington and Oregon coasts throughout our study (Mantua et al., 1997). Thus, it is unlikely that parent and offspring generations experienced differences in marine growth rates, which could have biased our heritability results. It is also possible that good marine growth conditions reduced selection pressures on length at maturity; however, we were unable to address this possibility with our dataset. An interesting future question would be whether favorable marine growth impacts selection on body size to the same extent in females, hooknose males, and jacks.

Comparing the degree of sexual and tactical conflict across salmonid species with different degrees of ART expression could provide a macroevolutionary perspective on the relationship between inter‐ and intraspecific variation and intralocus conflict. Male ARTs are common in Coho, Chinook, Atlantic salmon, and Steelhead trout, but less common in most populations of sockeye salmon, and nearly nonexistent in pink and chum salmon (Fleming, 1996; Quinn, 2018). One might expect that life‐history traits such as length at maturity would have no intersexual correlation in species where ARTs are common, a low intersexual correlation in species where ARTs are rare, and a high intersexual correlation where ARTs do not exist. Repeating these analyses across pedigrees from different salmonid species and over more generations could result in a more general theoretical model encompassing both inter‐ and intrasexual variation and conflict.

## CONFLICT OF INTEREST

The authors declare no conflicts of interest.

## Data Availability

The data that support the findings of this study are openly available in ScholarsArchive@OSU at https://doi.org/10.7267/N9H41PB9.
